# The safety of percutaneous renal biopsy for acute kidney injury in metastatic renal cell cancer patients with reduced nephron mass

**DOI:** 10.3389/fneph.2025.1615779

**Published:** 2025-08-06

**Authors:** Tomaz Milanez, Vinay Srinivasan, Vladimir Premru, Miha Arnol, Janja Ocvirk, Edgar A. Jaimes

**Affiliations:** ^1^ Division of Medical Oncology, Institute of Oncology Ljubljana, Ljubljana, Slovenia; ^2^ Department of Nephrology, University Medical Center Ljubljana, Ljubljana, Slovenia; ^3^ Division of Nephrology, Cooper University Hospital and Cooper Medical School of Rowan University, Camden, NJ, United States; ^4^ Faculty of Medicine, University of Ljubljana, Ljubljana, Slovenia; ^5^ Renal Service, Department of Medicine, Memorial Sloan Kettering Cancer Center, New York, NY, United States; ^6^ Weill Cornell Medical College, New York, NY, United States

**Keywords:** acute kidney injury, complications, renal biopsy, renal cell carcinoma, solitary kidney

## Abstract

**Background:**

Percutaneous renal biopsy (PRB) provides valuable information to guide treatment decisions in patients with metastatic renal cell carcinoma (mRCC) who develop acute kidney injury (AKI) after systemic anticancer therapy (SACT). The rising incidence of renal cell carcinoma (RCC) and the substantial impact of SACT on overall survival suggest a higher prevalence of RCC patients with reduced nephron mass and a solitary kidney (SK) requiring PRB for AKI. However, safety data on SK biopsies are scarce, and the potential for dialysis-requiring complications may deter clinicians.

**Methods:**

This retrospective case series reports the safety of 13 PRBs in 12 mRCC patients with reduced nephron mass who developed AKI during SACT as well as six PRBs in six patients with metastatic solid malignancies and AKI, which developed during SACT.

**Results:**

Eleven biopsies in mRCC patients and five biopsies in patients with metastatic solid malignancies were uneventful. One patient with mRCC experienced a major bleeding event due to an arteriovenous (AV) fistula seven days post-procedure, while another mRCC patient developed macrohematuria within 24 hours. In the group of patients with metastatic solid malignancies, one patient experienced a small perinephric hematoma during the observational period. Despite the small sample size, individual chart reviews and direct management of adverse events allowed assessment of the association between biopsy and complications.

**Conclusion:**

Until further data become available, a longer observation period is recommended for these patient cohorts compared to the general population. Further studies are needed to develop consensus guidelines for PRB in mRCC patients with reduced nephron mass.

## 1 Introduction

Percutaneous renal biopsy (PRB) is the gold standard for assessing renal parenchymal disease. An accurate histologic diagnosis is key to guiding further decision making in patients with metastatic renal cell carcinoma (mRCC) and acute kidney injury (AKI) who have received various systemic anticancer therapy (SACT), such as immune checkpoint inhibitors (ICIs) and vascular endothelial growth factor receptor tyrosine kinase inhibitors (VEGFR-TKI) (1, 2).

Up to 82% of patients with advanced renal cell carcinoma (RCC) undergo total or partial nephrectomy, resulting in reduced nephron mass before starting sequential SACT for metastatic disease (3–6). Furthermore, approximately 50% of patients with synchronous advanced disease who begin SACT with a tumor in place receive cytoreductive nephrectomy during their first-line therapy, followed by continued SACT (7). Localized clear cell renal cell carcinoma (ccRCC) patients frequently undergo radical nephrectomy before adjuvant therapy (up to 92%), and approximately 26% subsequently receive VEGFR-TKI or ICIs due to disease progression (8). The rising incidence of RCC, coupled with the substantial impact of ICI-based SACT on overall survival (OS) in both mRCC patients and localized disease patients, suggests a higher prevalence of RCC patients with reduced nephron mass who may require PRB due to AKI associated with SACT (9, 10).

Data on the safety of solitary kidney (SK) biopsies are limited, leading to clinician hesitance due to potential complications requiring nephrectomy. However, foregoing PRB can delay appropriate treatment or result in unnecessary initiation of corticosteroid therapy for suspected ICI induced interstitial nephritis, potentially impacting OS (2, 11).

While PRB of native kidneys is generally considered a low-risk procedure in the general population, bleeding complications remain a significant concern. Advanced age, diabetes, anemia, metastatic disease, liver disease, and pre-existing AKI all increase the risk of complications during native PRB (12–14). Although the incidence of major bleeding events or death is similar between SK and native kidney biopsies in the general population, the potential consequences of an ablative procedure to manage major bleeding in an SK patient can lead to dialysis dependence. Whether patients with a SK receiving SACT face a similar risk remains unclear. The increased risk of hypertension and bleeding associated with SACT further raises safety concerns about PRB in this patient group. To our knowledge, no published reports describe PRB use for AKI evaluation in patients receiving SACT for mRCC with reduced nephron mass.

## 2 Methods

### 2.1 Patient population

This retrospective analysis was performed at the Institute of Oncology Ljubljana and the University Medical Centre Ljubljana, Slovenia, and Cooper University Hospital, Camden, New Jersey, USA. Individual chart reviews analyzed complications associated with PRBs performed between 2018 and 2023 for the evaluation of AKI in mRCC patients with reduced nephron mass and non-mRCC PRBs were performed at least seven days after AKI detection. All patients received SACT with VEGFR-TKIs or ICIs before AKI development, and SACT was withheld for at least seven days before PRB. During the same period, we gathered data on complications of PRBs in patients with metastatic solid cancers who experienced AKI as an adverse event (AE) during SACT. One of those patients had a SK while the others did not have reduced renal parenchymal mass.

### 2.2 Definitions

Reduced nephron mass was defined as a condition following total or partial nephrectomy or the presence of a primary tumor. AEs, including AKI, proteinuria, hypertension, and anemia, were graded according to the Common Terminology Criteria for Adverse Events, version 5.0. Minor bleeding complications were defined as macroscopic hematuria or hematoma not requiring blood products. Major bleeding complications were defined similarly to other studies: the need for blood products, radiologic or surgical intervention, intensive care unit admission, loss of parenchymal functional mass, or death (15, 16).

### 2.3 Patient preparation

Informed written consent was obtained from all patients after discussing the risks and benefits of PRB. All patients underwent a thorough evaluation, including complete blood count, metabolic panel, and coagulation profile tests. Anemia was corrected according to protocol recommendations, which included a minimum hemoglobin level of 10 g/dl. Kidney ultrasound was performed pre-biopsy to visualize kidney anatomy and rule out obstruction as a cause of AKI.

Platelet aggregation inhibitors (aspirin, clopidogrel, non-steroidal anti-inflammatory drugs) were discontinued at least seven days prior to PRB unless contraindicated by vascular risk factors (17). In high-risk patients, a specialist consultation determined the appropriateness of antithrombotic therapy, potentially including low-dose aspirin (≤100 mg) (18).

For patients on anticoagulation, warfarin was discontinued five days prior to PRB, with an INR target of <1.5. Unfractionated heparin bridge therapy was used if needed and the infusion ceased six hours prior to PRB and resumed 12 hours post-biopsy. Patients receiving low-molecular-weight heparin received their last dose 12 hours prior to PRB, with anticoagulation restarted 12 hours later. Hypertensive patients had to maintain blood pressures below 150/90 mmHg before PRB.

### 2.4 Procedure

All PRBs were performed under ultrasound guidance by a skilled nephrologist, following the local biopsy protocol. Patients were positioned prone with a pillow under the upper abdomen to facilitate slight trunk flexion. Either a 16- or 18-gauge needle was used.

### 2.5 Post-biopsy monitoring

Post- procedure, patients remained supine position and refrained from strenuous activity. Pulse, blood pressure, and urine output were monitored regularly during a 24-hour observation period. Kidney ultrasound was performed to detect hematoma formation, and urine was monitored for gross hematuria. Hemoglobin concentrations were monitored as required by local protocol. Patients were discharged home 24 hours post-procedure if no complications were observed.

### 2.6 Statistical analysis

Descriptive statistics were used throughout the analysis. Categorical variables (patient characteristics and comorbidities, procedural details) were analyzed using absolute numbers and percentages. Due to the small sample size, numerical variables were summarized as medians with interquartile ranges (IQR; 25th and 75th percentiles). Microsoft Excel for Mac version 16.25 was used for data analysis.

## 3 Results

### 3.1 Patient characteristics

Thirteen PRBs were performed on 12 patients with mRCC and reduced nephron mass who developed AKI during SACT. Most patients (9/12) had ccRCC, and ten had previously undergone total nephrectomy. The baseline characteristics of the mRCC patients, including comorbidities, are shown in **Table 1**.

**Table 1:** Characteristics of patients with mRCC (n=12).

Age at RCC diagnosis, years, median (IQR)	64 (57-66)
Sex: male/female	9 (75)/3 (25)
Histology: clear cell/papillary/chromophobe	9 (75)/2 (16.7)/1 (8.3)
Total nephrectomy	10 (83.3)
Right/left	5 (41.7)/5 (41.7)
Partial nephrectomy (left)	1 (8.3)
Primary tumor in place (right)	1 (8.3)
Arterial hypertension	9 (75)
Type 2 diabetes mellitus	5 (41.7)
Dyslipidemia	8 (66.7)
Obesity	6 (50)
Ischemic heart disease	1 (8.3)
Venous thromboembolism	1 (8.3)
Prior malignant cardiac tamponade	1 (8.3)
CKD	6 (50)
Prior AKI	2 (16.7)^a^

All values (except age) indicate the number of patients (%).

AKI, acute kidney injury; CKD, chronic kidney disease; IQR, interquartile range; mRCC, metastatic renal cell carcinoma.

a Two patients experienced acute kidney injury stage ≥2 according to KDIGO criteria.

The median baseline estimated glomerular filtration rate (eGFR; CKD-EPI) before SACT initiation was 60.5 mL/min/1.73 m² (IQR, 42-70). One patient began SACT with an eGFR of 15 mL/min/1.73 m². **Table 2** details SACT regimens, AKI characteristics at PRB, and related AEs. In six cases of PRB, patients had grade (G)2 AKI. Two patients had G3 AKI, which was related to ICIs. Four patients had G3 proteinuria, which was linked to VEGFR-TKI therapy in three patients. Six patients had proteinuria ≥1 g/day, and two had clinical evidence of nephrotic syndrome. Three patients treated with ICIs also experienced extra-renal serious AEs related either to the immunotherapy itself or to the associated therapy.

**Table 2:** Baseline SCr before sequential SACT, AKI-related characteristics at time of biopsy, and related measures in patients with mRCC (n=12).

No. of patient	No. of PRB	BaselinesCr μmol/L (mg/dL)	SACT at AKI	AKI G at PRB	Peak sCr at time of AKI μmol/L (mg/dL)	Proteinuria G3 at time of AKI	Proteinuria ≥1 g/day at time of AKI	Histological findings at time of AKI	Targeted management of AKI and SAEs aligned with CTG	Other SAEs during AKI
1	1	108 (1.22)	VEGFR-TKI (sunitinib)^a^	G2	237 (2.68)	Yes	Yes	AVTMA, MGN (IgG deposits), AIN, ATI, DN, HTN-NS	Permanent discontinuation of sunitinib	None
2	2	123 (1.39)	ICI (PD-1)^b^	G2	258 (2.92)	No	No	AIN, TBMD	Permanent discontinuation of ICI	AH
2	3	123 (1.39)	VEGFR-TKI (cabozantinib)^c^	G2	279 (3.16)	Yes	Yes	CGTMA, FSGS, ATI	Permanent discontinuation of cabozantinib	NS
3	4	87 (0.98)	ICI (PD-1)^d^	G2	260 (2.94)	No	Yes	ATI, TBMD, TMA-like arteriopathy	Permanent discontinuation of SACT	None
4	5	121 (1.37)	ICI (PD-1)^a^	G1	201 (2.27)	No	Yes	Glomerular mesangial and segmental parietal IgG deposits, HTN-NS, DN-Kimmelstiel Wilson	Permanent discontinuation of ICI	Colitis
5	6	129 (1.46)	ICI (PD-1)^a^	G1	238 (2.69)	No	No	HTN-NS, DN, ORG	None	None
6	7	116 (1.31)	ICI (PD-1)^b^	G2	240 (2.71)	Yes	Yes	DN-Kimmelstiel Wilson, modest IFTA	None	None
7	8	50 (0.57)	ICIs (CTLA-4 + PD-1)^a^	G3	396 (4.48)	No	No	AIN, ATI	Permanent discontinuation of ICI	CLS
8	9	289 (3.27)	ICI (PD-1)^b^	G1	341 (3.86)	No	No	HTN-NS, FSGS, TIN, moderate IFTA	None	None
9	10	163 (1.84)	VEGFR-TKI (cabozantinib)^c^	G1	263 (2.97)	Yes	Yes	CGVTMA, ATI, moderate IFTA	Permanent discontinuation of cabozantinib	NS, AH
10	11	108 (1.22)	ICIs (CTLA-4 + PD-1)^a^	G2	320 (3.62)	No	No	ATI, mild IFTA, FGGS	Permanent discontinuation of ICI	Colitis, encephalitis, sepsis
11	12	137 (1.55)	ICIs (CTLA-4 + PD-1)^a^	G3	420 (4.75)	No	No	ATI, signs of obstructive nephropathy	Discontinuation of ICI	UIB, urosepsis, urinary bleeding
12	13	84 (0.95)	ICIs (CTLA-4 + PD-1)^a^	G1	197 (2.23)	No	No	PAS positive hyaline casts in tubules but otherwise normal parenchyma, minimal IFTA	None	None

AH, arterial hypertension; AIN, acute interstitial nephritis; AKI, acute kidney injury; ATI, acute tubular injury; AVTMA, acute vascular thrombotic microangiopathy; CGTMA, chronic glomerular thrombotic microangiopathy; CGVTMA, chronic glomerular vascular thrombotic microangiopathy; CLS, capillary leak syndrome; CTG, cancer treatment goals; CTLA-4, cytotoxic T-lymphocyte-associated protein 4; DN, diabetic nephropathy; FGGS, focal and global glomerulosclerosis; FSGS, focal segmental glomerular sclerosis; G, grade of adverse event according to the Common Terminology Criteria for Adverse Events, version 5.0; IFTA, focal interstitial fibrosis and tubular atrophy; HTN-NS, hypertensive nephrosclerosis; ICI, immune checkpoint inhibitor; MGN, membranous glomerulonephritis; mRCC, metastatic renal cell carcinoma; NS, nephrotic syndrome; PD-1, programmed death 1; PRB, percutaneous renal biopsy; SACT, systemic anticancer therapy; SAEs, serious adverse events; sCr, serum creatinine; TBMD, thin basement membrane disease; TIN, tubulointerstitial nephrocalcinosis; TMA-thrombotic microangiopathy; UIB, upper intestinal bleeding; VEGFR-TKI, vascular endothelial growth factor receptor tyrosine kinase inhibitor.

a 1st line of SACT as VEGFR-TKI inhibitor; combination ICIs with CTLA-4 + PD-1 inhibitors (ipilimumab+nivolumab); maintenance therapy with ICI PD-1 inhibitor (nivolumab).

b 2nd line of SACT.

c 3rd line of SACT.

d 7th line of SACT.

The median patient age at PRB was 68 years (IQR, 60-72), the median serum creatinine was 2.17 mg/dL (IQR, 2.04-2.75), and the median hemoglobin level was 10.8 g/dL (IQR, 10.6-11.8). Platelet counts were within normal limits. One patient required a red blood cell transfusion before PRB, following standard protocol.

**Table 3** displays clinical characteristics and laboratory values at PRB of mRCC patients and patients with metastatic solid malignancies. **Table 4** lists medications administered prior to PRB that could increase the risk of bleeding.

**Table 3:** Clinical characteristics and laboratory values at PRB in mRCC patients (n=13) and patients with metastatic solid malignancies (n=6).

	mRCC patients	Patients with metastatic solid malignancies
No. of PRBs	13	6
Left kidney biopsy	6 (46.2)^a^	6
Right kidney biopsy	7 (53.8)^b^	0
Solitary kidney biopsy	11 (84.6)	1 (16.7)
Arterial hypertension >160/100 mmHg two hours prior to PRB	2 (15.4)^c^	None
Creatinine, mg/dL μmol/L	2.17 (2.04-2.75) 191.9 (180.4-243.2)	2.57 (1.93-3.23) 227 (170.5-285.5)
Urea, mmol/L	14 (11-16)	18 (12-24)
Hemoglobin, g/dL	10.8 (10.6-11.8)	10.9 (10.6-11.8)
Bleeding time, s	102 (96-110)^d^	25^l^
Prothrombin time, INR	1.04 (0.94-1.13)	1.1 (1.05-1.15)
aPTT, s	32 (30-34)^e^	27 (25-33)
Platelet count, x 10^9^/L	209 (153-269)	255 (166-260)
The highest level of proteinuria during AKI, g/day	0.50 (0.21-4.92)^f^	0.30 (0.21-1.20)^m^
The last measured proteinuria value prior to PRB, g/day	0.21 (0.18-2.00)	0.35 (0.15-0.95)
Use of an 18-gauge needle	12 (92.3)^g^	6
No. of needle passes	3 (2-4)	2 (2-2)
Depth of the biopsied kidney, cm	7.0 (5.5-9.0)^h^	5.4 (4.5-6.7)
Bipolar diameter of the biopsied kidney, cm	11.5 (13.2-13.2)	11.5 (11.0-12.3)
Parenchymal thickness, cm	1.8 (1.5-2.0)^i^	1.7 (1.5-2.0)
Acquired kidney cysts at the PRB in the biopsied kidney	4 (30.8)^j^	None
BMI ≥30 kg/m^2^	6 (46.2)^k^	None

Values for continuous variables are given as median (IQR); values for categorical variables indicate the number of biopsies (%).

AKI, acute kidney injury; aPTT, activated partial thromboplastin time; BMI, body mass index; INR, international normalized ratio; IQR, interquartile range; mRCC, metastatic renal cell carcinoma; PRB, percutaneous renal biopsy.

a A left kidney PRB was performed due to a tumor in place in the right kidney.

b A right kidney PRB was performed due to a small left kidney from prior partial nephrectomy.

c Two patients required additional anti-hypertensives due to poorly controlled hypertension.

d In six cases bleeding time was not measured.

e In three cases aPTT was not measured.

f In one case without clinical signs of proteinuria data about the highest level of proteinuria at AKI is missing.

g In one case a 16-gauge needle was used.

h In one case depth of the biopsied kidney was not measured.

i In one case parenchymal thickness was not measured.

j No. >1 and/or diameter >1 cm of acquired kidney cysts.

k One patient with the right kidney at a depth of 11.5 cm, and one patient with the left kidney at a depth of 12 cm.

l In one case bleeding time was measured.

**Table 4:** Use of medications prior to PRB that may increase the risk of bleeding in mRCC patients (n=13) and patients with metastatic solid malignancies (n=6).

Medications	No. of PRBs (%) in mRCC patients	No. of PRBs (%) in patients with metastatic solid malignancies
AT (aspirin + clopidogrel)	1 (7.7)^a^	None
AT (aspirin)	1 (7.7)^b^	1 (16.7)^f^
Anticoagulant therapy	2 (15.4)^c^	None
Corticosteroid therapy	5 (38.5)^d^	5 (83.3)^g^
VEGFR-TKIs	4 (30.8)^e^	1 (16.7)^h^
BRAFi and MEKi	None	1 (16.7)^i^

AT, antiplatelet therapy; BRAFi, BRAF (V600) protein kinase inhibitor; MEKi, mitogen-activated protein kinase inhibitor; mRCC, metastatic renal cell carcinoma; PRB, percutaneous renal biopsy; VEGFR-TKIs, vascular endothelial growth factor receptor tyrosine kinase inhibitors.

a In one patient dual AT due to cerebrovascular disease was held one week prior to PRB.

b Patient with ischemic heart disease at very high risk for vascular events was continued on monotherapy with aspirin at a low dose of 100 mg. This patient had a solitary kidney, the PRB carried additional risk due to the anatomical position of the solitary kidney.

c One patient continued therapy with low molecular weight heparin and the other patient’s warfarin was switched to unfractionated heparin before the procedure.

d Four patients received corticosteroid therapy at the time of PRB due to immune-related AEs and three of the four received high dose of corticosteroids (≥1 mg/kg/day).

e Four patients were receiving VEGFR-TKI therapy, which was discontinued at least 14 days prior to PRB.

f In one patient AT due to peripheral arterial occlusive disease and ischemic heart disease was held one week prior to PRB.

g Five patients received corticosteroid therapy at the time of PRB due to immune-related AEs and four of the five received high dose of corticosteroids (≥1 mg/kg/day).

h One patient was receiving VEGFR-TKI therapy, which was discontinued at least 14 days prior to PRB.

i One patient was receiving combination BRAFi and MEKi therapy, which was discontinued at least 14 days prior to PRB.

Six PRBs were performed in six patients with metastatic solid cancers who had developed AKI during SACT. Two patients had melanoma, two had urothelial cancer, one had thyroid cancer, and one had gastric cancer. Except for one patient with a SK following right nephrectomy for upper urinary tract carcinoma, none of the patients met the criteria for reduced renal mass. All patients with metastatic solid cancer began SACT with eGFR >50 mL/min/1.73 m².

Among patients with metastatic solid cancers undergoing PRB, two had G4 AKI, one of them had a SK, and both underwent acute hemodialysis prior to biopsy. Three patients experienced G3 AKI, and one patient had G2 AKI. A patient with thyroid cancer developed nephrotic syndrome during VEGFR-TKI therapy, with peak proteinuria at 6.85 g/day during G3 AKI. One patient required a red blood cell transfusion before PRB.

In the cohort of six patients with metastatic solid malignancies, the median patient age at PRB was 63 years (IQR, 53.5–65.5), the median hemoglobin level was 10.9 g/dL (IQR, 10.6–11.8), and the median platelet count was 255 x 10^9^/L (IQR, 166-260).

### 3.2 Complications related to PRB

One mRCC patient with a SK and grade 3 AKI (due to ICIs) developed an arteriovenous (AV) fistula, detected by post-procedure kidney ultrasound. Seven days later, genitourinary hemorrhage caused hemodynamic instability requiring emergent AV fistula coiling and a red blood cell transfusion. The patient’s course was further complicated by upper gastrointestinal bleeding and urosepsis. Prior to biopsy, this patient had not received antithrombotic therapy but had been exposed to high-dose corticosteroids. Despite the severity of the complications, the patient fully recovered.

Another mRCC patient with a SK, grade 1 AKI and nephrotic syndrome due to cabozantinib required a red blood cell transfusion pre-biopsy, having remained on aspirin. Gross hematuria developed during the observation period, but no intervention was necessary. No other patients experienced procedure-related complications.

During the observational period, a patient with metastatic urothelial cancer, who had both kidneys, experienced a minor bleeding event after a post-procedure ultrasound revealed a 15 mm perirenal hematoma in the left kidney. However, no further measures were necessary. The patient had been exposed to high-dose corticosteroids due to AEs prior to biopsy. Antiplatelet therapy with low-dose aspirin had been discontinued seven days prior to the procedure.

## 4 Discussion

This study assessed PRB safety in mRCC patients with reduced nephron mass receiving SACT. This is, to our knowledge, the first safety analysis of PRB in this specific population. Although clinicians are often hesitant to perform PRB in patients with a SK, there are an increasing number of individuals with mRCC and a SK who require a comprehensive evaluation for AKI or proteinuria. Due to the incidence and prevalence of high-grade AKI in this patient population, there is a critical need for an accurate and timely biopsy evaluation to avoid a negative impact on planned treatment goals.

A cross-sectional study in Australia reported that nephrologists are less likely to perform PRBs in SK patients (19). However, limited data exist regarding PRB complications in SK patients. One prospective registry showed that eight of nine SK patients underwent successful PRB, with gross hematuria occurring in only one patient (20). A large retrospective study (n>118,000) showed a high red blood cell transfusion rate (26%), although not all complications were directly attributed to biopsy. Cohort heterogeneity regarding cancer diagnosis and staging limited meaningful subgroup analysis of SK patients (21).

AV fistula complications post-PRB vary widely (0.7-15%), though hemodynamic instability is rare. The difference in complication rates compared to other studies could be because Sosa-Barrios et al. used routine post-biopsy ultrasound and Doppler imaging by nephrologists trained in ultrasound (22). In contrast, another multicenter study showed that 26% of biopsies were performed by non-nephrologists without routine post-biopsy imaging, resulting in a 5% major complication rate and a 22% increased risk per additional needle pass. This study also suggested a protective effect of high proteinuria (odds ratio 0.95 per additional g/day) against major complications, with a median proteinuria of 2.4 g/day and 38.5% of patients exhibiting nephrotic-range proteinuria (15). In our cohort, both patients experiencing bleeding complications had two passes; one patient required four passes yet experienced no complications.

In our cohort of mRCC patients, five of 13 biopsies (38.4%) showed proteinuria ≥1 g/day; four (30.8%) had nephrotic range proteinuria; and two patients exhibited clinical evidence of nephrotic syndrome. Median proteinuria before PRB was 0.21 g/day (IQR, 0.18-2.0), and the highest median during AKI was 0.5 g/day (IQR, 0.21-4.92). The difference likely resulted from VEGFR-TKI withdrawal prior to biopsy. In the cohort of six patients with metastatic solid malignancies, the median value of proteinuria before PRB was 0.35 g/day (IQR, 0.15-0.95). Only one patient experienced proteinuria ≥1 g/day with a peak level of 6.85 g/day detected prior to PRB, along with nephrotic syndrome due to the VEGFR-TKI.

A meta-analysis showed a higher rate of red blood cell transfusions with 14-gauge (2.1%) versus 16-gauge (0.4%) or 18-gauge (0.6%) needles (13). Sixteen-gauge needles represent a reasonable compromise, and needles smaller than 16-gauge should be avoided (23). We used 18-gauge needles for all three patients with bleeding complications (two with mRCC and one with metastatic solid cancer), neither of whom had obvious procedural or anatomical risk factors.

The increased bleeding risk in cancer patients treated with certain VEGFR-TKIs (sunitinib, bevacizumab, sorafenib) necessitates careful consideration of anticoagulant risk factors (24, 25). In our cohort, VEGFR-TKI therapy was discontinued at least two weeks prior to PRB. Existing guidelines generally recommend discontinuing antithrombotic therapy 7–10 days before elective PRB, but in urgent cases or high-risk cardiovascular patients, low-dose aspirin monotherapy (≤100 mg) may be considered safe (17). One patient in our cohort, on aspirin monotherapy (100 mg), experienced gross hematuria. A meta-analysis showed no significant association between aspirin use and serious bleeding complications, although the definition of aspirin exposure varied across studies (26).

A prospective observational study of native kidney biopsies in patients with eGFR <30 mL/min/1.73 m² found a 5.6% major complication rate, excluding SK patients. Patients with eGFR <15 mL/min/1.73 m² had a significantly higher rate of hematomas ≥2 cm (27). Our cohort of mRCC patients had a median baseline eGFR of 60.5 mL/min/1.73 m² (IQR, 42-70) with one SK patient beginning SACT at 15 mL/min/1.73 m². This patient, however, experienced no complications. Among six patients with metastatic solid malignancies nobody began SACT with eGFR <50 mL/min/1.73 m².

Well-controlled hypertension does not appear to increase biopsy risk (28) while uncontrolled hypertension (≥160/100 mmHg) is a relative contraindication (17). Two mRCC patients required antihypertensive medication before PRB due to blood pressures exceeding 160/90 mmHg but did not experience post-biopsy bleeding complications.

In the cohort of mRCC patients, eight of thirteen (61.5%) biopsies were performed in patients with G2 or higher AKI (including two G3 cases), at least seven days after peak creatinine levels. In the cohort of patients with metastatic solid malignancies, two required acute hemodialysis due to AKI; one patient with SK remained on renal replacement therapy during the period of PRB. A systematic review and meta-analysis of over 100,000 native kidney biopsies showed that hospitalized patients with AKI have a higher risk of post-biopsy complications (29). A single-center study of patients with acute kidney disease, primarily AKI, also demonstrated increased risk in hospitalized patients, excluding those with renal malignancy. In that study, 8% of hospitalized patients with acute kidney disease required red blood cell transfusions, and 2% needed interventions to stop bleeding, compared to none in the non-hospitalized group (30). In our cohorts, neither patient experiencing bleeding complications was hospitalized, and all underwent elective biopsies at least seven days post AKI onset.

Historically, patients were observed for 24 hours post-biopsy to monitor for procedure-related complications (31). An earlier study showed that 77% of complications were apparent by eight hours and 100% of serious complications by 24 hours (32). While this study used real-time ultrasound, not all used automated biopsy needles (32). In his retrospective analysis, Abuelo recommended a 6–8-hour observation period while other studies suggest 8–12 hours for uncomplicated biopsies, but 24 hours for high-risk patients or those living far from a hospital (17, 33).

Based on our findings and previous studies, we recommend 24-hour post-biopsy observation for this specific patient population. Routine post-biopsy ultrasound is also recommended. The patient with a major complication (hemodynamically significant AV fistula) had no other significant risk factors. The patient with gross hematuria was on low-dose aspirin monotherapy due to cardiovascular risk. In a patient with small perinephric hematoma, low-dose heparin monotherapy due to ischemic heart disease and peripheral artery disease was withdrawn seven days before the PRB. While some studies suggest an increased risk of complications with aspirin use, this is not consistently observed (17, 26).

Our retrospective analysis has several strengths. Chart review and direct AE management allowed assessment of the causal relationship between biopsy and complications. The homogeneous studied cohort (mRCC, reduced nephron mass, AKI during SACT) allowed meaningful comparison to other similar studies, though the small sample size limits generalizability.

## 5 Conclusions

Renal biopsy is essential when histopathology could significantly impact treatment, but it is challenging in SK patients due to potential complications. This study, using a homogeneous mRCC cohort in a real-world clinical setting, reveals a potential link between PRB and safety outcomes. Given the lack of safety data, a longer post-PRB observation period (24 hours) is prudent for mRCC patients with reduced nephron mass and AKI receiving SACT. Patients should be fully informed about the limited safety data. Due to high survival rates in mRCC patients, the associated nephrotoxic risks of SACT, and the potential for biopsy-related complications, large prospective observational studies are needed. Consensus guidelines for PRB in this patient population are crucial to standardize care and improve outcomes.

## Data Availability

The raw data supporting the conclusions of this article will be made available by the authors, without undue reservation.
